# Left atrial appendage flow velocities: assessment by velocity encoded magnetic resonance imaging

**DOI:** 10.1186/1532-429X-13-S1-P252

**Published:** 2011-02-02

**Authors:** Kai Muellerleile, Arian Sultan, Michael Groth, Daniel Steven, Imke Drewitz, Boris Hoffmann, Gerhard Adam, Gunnar K Lund, Thomas Rostock, Stephan Willems

**Affiliations:** 1 University Medical Center Hamburg-Eppendorf, Hamburg, Germany

## Purpose

To evaluate the use of velocity encoded (VENC) magnetic resonance imaging (MRI) for measurements of left atrial appendage (LAA) flow velocities in comparison with transesophageal echocardiography (TEE).

## Introduction

The presence of reduced LAA flow velocities indicates patients who are prone to thrombus formation in the LAA and therefore being at high risk for subsequent cardioembolic stroke. LAA flow velocities are typically assessed by TEE in clinical routine. VENC-MRI is an established tool for the quantification of transvalvular flow, but the feasibility of LAA flow measurements by VENC-MRI has not been studied so far.

## Methods

**Figure 1:**
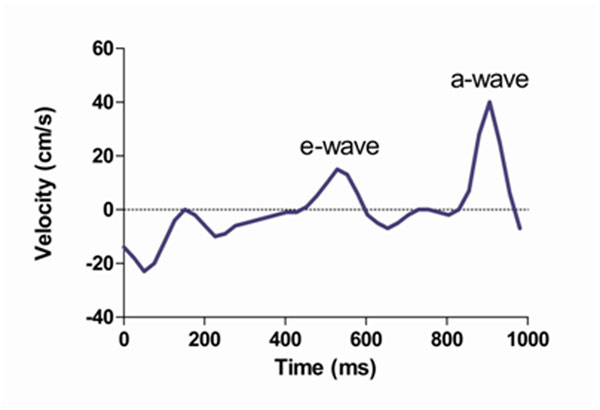
Measurements of LAA flow velocities by VENC-MRI

The study included 26 patients with sinus rhythm (n = 15) or atrial fibrillation (n = 11). All patients underwent VENC-MRI and TEE to assess LAA flow velocities. VENC-MRI was performed perpendicular to the orifice of the LAA to assess through-plane flow. Peak velocities were measured of the passive, early-diastolic LAA outflow (e-wave) in all patients. Peak velocities of active, late-diastolic LAA outflow (a-wave) were assessed in patients with sinus rhythm. Figure 1 demonstrates measurements of LAA flow velocities by VENC-MRI in a patient with sinus rhythm. Correlation and agreement was analyzed between VENC-MRI and TEE measurements of e- and a-wave peak velocities and the e/a ratio.

## Results

A good correlation and agreement was found between VENC-MRI and TEE measurements of maximal e-wave velocities (r = 0.63, P<0.001; mean difference 1±10 cm/s). The a-wave was detected by VENC-MRI in all patients with sinus rhythm. Correlation was strong for measurements of maximal a-wave velocities between VENC-MRI and TEE (r=0.73, P<0.01). A mean difference of 10±16 cm/s was found between VENC-MRI and TEE maximal a-wave velocities. There was a good correlation and agreement for the e/a ratio between VENC-MRI and TEE (r=0.63, P<0.01, mean difference 0.08±0.12).

## Conclusions

The assessment of LAA flow velocities by VENC-MRI is feasible and measurements agree well with TEE. VENC-MRI has the potential to identify patients who are at risk for thrombus formation in the LAA.

